# Non-canonical Wnt signals regulate cytoskeletal remodeling in osteoclasts

**DOI:** 10.1007/s00018-018-2881-1

**Published:** 2018-07-26

**Authors:** Shunsuke Uehara, Nobuyuki Udagawa, Yasuhiro Kobayashi

**Affiliations:** 1 Department of Biochemistry, Matsumoto Dental University, Nagano, 399-0781 Japan; 2 Institute for Oral Science, Matsumoto Dental University, 1780 Gobara, Hiro-oka, Shiojiri, Nagano 399-0781 Japan

**Keywords:** Actin, Bone resorption, Osteoclast, Rho effectors, Wnt non-canonical pathway

## Abstract

Osteoclasts are multinucleated cells responsible for bone resorption. Osteoclasts adhere to the bone surface through integrins and polarize to form actin rings, which are formed by the assembly of podosomes. The area contained within actin rings (also called sealing zones) has an acidic pH, which causes dissolution of bone minerals including hydroxyapatite and the degradation of matrix proteins including type I collagen by the protease cathepsin K. Osteoclasts resorb bone matrices while moving on bone surfaces. Osteoclasts change their cell shapes and exhibit three modes for bone resorption: motile resorbing mode for digging trenches, static resorbing mode for digging pits, and motile non-resorbing mode. Therefore, the actin cytoskeleton is actively remodeled in osteoclasts. Recent studies have revealed that many molecules, such as Rac, Cdc42, Rho, and small GTPase regulators and effectors, are involved in actin cytoskeletal remodeling during the formation of actin rings and resorption cavities on bone slices. In this review, we introduce how these molecules and non-canonical Wnt signaling regulate the bone-resorbing activity of osteoclasts.

## Introduction

Bone is continually remodeled by bone-forming osteoblasts and bone-resorbing osteoclasts [1–4]. The balance between bone formation and resorption maintains bone mass. An imbalance between bone resorption and formation leads to bone metabolic diseases, including osteoporosis [1–4]. Osteoporosis occurs when bone resorption is higher than bone formation. Similarly, when bone formation is higher than bone resorption, bone mass increases. Estrogen deficiency enhances bone resorption, which, in turn, reduces bone mass in postmenopausal osteoporosis [3]. Inflammatory cytokines, such as TNF-α, IL-6, and IL-17, induces bone destruction by osteoclasts in inflammatory diseases such as rheumatoid arthritis and periodontal disease [4, 5]. Antiresorptive agents have two pharmacological actions, inhibition of osteoclast formation and inhibition of the bone-resorbing activity of osteoclasts [6–8]. An anti-receptor activator of NF-κB ligand antibodies (denosumab) inhibits osteoclast differentiation [6, 7]. Bisphosphonates suppress the bone-resorbing activity of osteoclasts [6, 8]. A deeper understanding of the mechanisms by which osteoclasts resorb bone may lead to the development of new antiresorptive drugs.

Osteoclasts are one of the cells, in which actin cytoskeleton is highly organized. Recent studies have established roles of actin cytoskeletons in cell functions such as cell migration, adhesion, mitosis, and sensing the external environment [9–11]. Polymerization and depolymerization of globular-actin reorganize the cytoskeleton and are required for cell movement through the formation of lamellipodia and filopodia [9, 10, 12, 13]. When cells contact the extracellular matrix (ECM), focal adhesions and podosomes are formed at the adhesion site, and the cytoskeleton becomes organized [10, 14, 15]. Podosomes are unique structures in osteoclasts, invasive cancer cells, vascular smooth muscle cells, endothelial cells, and myeloid cells such as macrophages and dendritic cells [14–18]. Podosomes are also formed in fibroblasts expressing a constitutively active form of the non-receptor tyrosine kinase c-Src or v-Src, indicating that critical roles of active c-Src in podosome formation [19, 20]. Osteoclasts strongly express c-Src, and *c*-*Src*-deficient osteoclasts exhibited impaired bone-resorbing activity with defects of the formation of actin rings, ring-like structures of podosomes [21]. Thus, an understanding of signals that activate c-Src may lead to the discovery of new therapeutic targets for bone diseases such as osteoporosis and periodontal disease.

In this review, we introduce important findings on how actin rings are regulated by signals from integrins and small GTPases to exert bone-resorbing activity of osteoclasts and discuss how non-canonical Wnt signals regulate bone-resorbing activity of osteoclasts through small GTPases.

## Osteoclasts

**Fig. 1:**
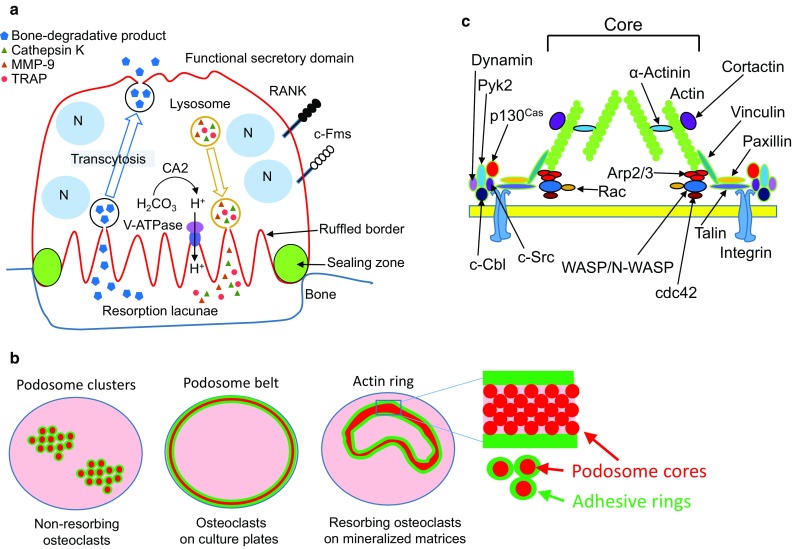
**a** Schematic representation of a bone-resorbing osteoclast. Osteoclasts adhere to bone and are polarized. Tartrate-resistant acid phosphatase (TRAP), H^+^, Cl^−^, and proteolytic enzymes, such as MMP-9 and cathepsin K, are secreted from the ruffled border, where the plasma membrane contacts the bone surface to degrade bone. Bone degradation products pass through osteoclasts by transcytosis and are released from functional secretory domain, opposite the bone surface. **b** Schematic representation of podosome clusters (left), a podosome belt (middle), and an actin ring (right) [69]. Red circles indicate podosome core. Green circles indicate adhesive rings, and green area means that proteins constituting the adhesive rings are localized. **c** Schematic representation of a podosome. Elongation of F-actin occurs in the core of the podosome. The peripheral region of the core includes the adhesion molecule integrin, c-Src, Pyk2, and adapter proteins such as paxillin, talin, and vinculin [29]

Osteoclasts are multinucleated cells responsible for bone resorption (Fig. 1a) and differentiate from monocyte–macrophage-lineage progenitor cells [22–24]. Two cytokines, macrophage colony-stimulating factor (M-CSF, also called CSF-1) and receptor activator of nuclear factor-κB (RANK) ligand (RANKL), are essential for osteoclast differentiation [22–24]. Osteoclast precursors express RANK and the M-CSF receptor c-Fms. When M-CSF and RANKL bind to c-Fms and RANK, respectively, osteoclast precursors differentiate into osteoclasts.

Osteoclasts change their cell shapes and exhibit the following states: motile resorbing osteoclasts [25, 26], static resorbing osteoclasts, and motile non-resorbing osteoclasts [27]. When filamentous (F)-actin in osteoclasts cultured on mineralized matrices such as bone slices is stained with rhodamine-labeled phalloidin, ring-like structures of F-actin dots (therefore called actin rings) are observed in osteoclasts under fluorescent microscopy (Fig. 1b, right). Actin rings are also called sealing zones, which shield the extracellular space surrounded by actin rings (hereafter called Howship’s lacunae) from the environment [24, 28, 29]. The Howship’s lacunae are acidified by secreted protons and chloride ions to dissolve bone mineral. Carbonic anhydrase II catalyzes rapid inter-conversion of carbon dioxide and water to bicarbonate, carbonic acid, and protons. Protons and chloride ions are secreted into Howship’s lacunae via a vacuolar-type H^+^-ATPase and chloride channel 7, respectively [24, 28, 29]. Proteases, such as cathepsin K and MMP-9, are also secreted into Howship’s lacunae and degrade type I collagen, a major matrix protein in bone. The plasma membrane in contact with Howship’s lacunae exhibits ruffled borders through massive fusion of lysosome-like vesicles. Degradation products of bone matrices are taken up into osteoclasts and secreted from the functional secretory domain in their basolateral membrane. This process is called transcytosis [30, 31]. Thus, organization of actin cytoskeletons in osteoclasts is necessary to keep acidic in Howship’s lacunae to resorb bone.

Bone-resorbing activity of osteoclasts was detected in vivo and in vitro experiments. The C-terminal telopeptide of type I collagen is known for a useful serum marker for bone resorption, and widely used in patients and animal models with bone metabolic diseases. In vitro experiments, detection of resorption pits is a useful method for accessing resorbing activity of osteoclasts [32]. Furthermore, we have reported that a part of the resorption lacunae is positive for tartrate-resistant acid phosphatase (TRAP) activity, and osteoclasts with actin rings are just present on TRAP activity-positive resorption lacunae, indicating that polarized osteoclasts secrete TRAP into resorption lacunae [33]. Thus, formation of resorption pits and actin rings in vitro is indicating that osteoclasts are polarized and resorb bone.

## Podosomes

Podosomes are actin-rich, adhesive structures observed in several kinds of cells including osteoclasts, and are important for rigidity sensing, bone resorption, antigen sampling, and formation of invadopodia [14, 15, 17, 28, 34–36]. A better understanding of podosome structures helps to clarify how podosomes are remodeled in osteoclasts stimulated by various signals including Wnt signals. Here, phenotypes of osteoclasts formed from mice lacking the genes encoding proteins constituting podosomes are introduced.

The state of podosomes is dependent on both the activity of osteoclasts and ECM. Bone-resorbing osteoclasts have actin rings. In contrast, non-resorbing osteoclasts without actin rings have podosome clusters in their cytoplasm (Fig. 1b, left). Osteoclasts cultured on plastic plates have a belt-like structure of podosomes called the podosome belt at the periphery of cells (Fig. 1b, middle), indicating that ECM affects the distribution of podosomes in osteoclasts. Osteoclasts cultured on plastic plates can secrete acid comparable to osteoclasts on bone [37]. Therefore, there may not be a large functional difference between actin rings and podosome belts.

Osteoclasts have been considered to stop arbitrarily and form actin rings to resorb bone. Two phases of mature osteoclasts were observed using intravital multiphoton microscopy: static resorbing osteoclasts and motile non-resorbing osteoclasts [27]. However, it has recently been reported that osteoclasts move laterally on the bone surface and generate long trench without disassembling and reconstructing podosomes [25, 26]. Thus, osteoclasts resorb bone under either two states such as pit or trench resorption modes (hereafter, resorption pits and trenches are simply called resorption cavities unless, otherwise, distinguished). It should be clarified how these modes are switched in osteoclasts in future.

Podosomes are composed of a large number of proteins and can be divided into an actin-rich protrusive core and an adhesive ring around the core ([29], Fig. 1b, c). The adhesive ring includes the adhesion molecule integrin, adaptor proteins, the tyrosine kinase c-Src, and proline-rich tyrosine kinase 2 (Pyk2). The adapter proteins paxillin, talin, and vinculin connect integrin and actin. The podosome core contains Wiskott–Aldrich syndrome protein (WASP), neural-WASP (N-WASP), and the Arp2/3 complex, and is the site where F-actin elongates [38–41]. Cortactin is also in the podosome core and stabilizes F-actin [42].

Osteoclasts deficient in *c*-*Src, Pyk2, Wasp, Cortactin, Talin*, or *Vinculin* have impaired bone-resorbing activity in vitro [43–48]. *c*-*Src*-deficient mice have osteopetrosis from impaired bone resorption [43]. Formation of actin rings and resorption cavities is markedly impaired in *c*-*Src*-deficient osteoclasts [21, 44]. *Pyk2*-deficient mice also have increased bone mass from the suppression of bone-resorbing activity of osteoclasts [45]. *Pyk2*-deficient osteoclasts do not form actin rings because of the reduced stability of microtubules from excessive Rho activation [45], suggesting that excess activation of Rho inhibits the podosome formation.

The bone mass in *c*-*Src*-deficient mice exhibited higher than that in *Pyk2*-deficient mice, even though c-Src forms a complex with Pyk2 for the podosome formation [46]. Furthermore, mice with either osteoclast-specific *Talin1* deficiency or an osteoclast precursor-specific *Vinculin* deficiency showed increased bone mass, but failed to show osteopetrosis as shown in *c*-*Src*-deficient mice [47, 48]. These findings suggest that c-Src has other important roles in osteoclast function in addition to its role in podosomes.

Outside-in signals from integrins activate c-Src to form actin rings in osteoclasts. To explore the roles of integrins in bone resorption, *β3 integrin*-deficient mice have been generated and studied, because osteoclasts strongly express αvβ3 integrins [49, 50]. *β3 integrin*-deficient mice developed osteopetrotic phenotype due to defects in bone-resorbing activity of osteoclasts [51]. Unlike *c*-*Src*-deficient mice, the osteopetrotic phenotype was developed 3–6 months after birth. This finding also suggests that c-Src is involved in osteoclast functions other than podosome formation and that other integrins complement the functions of β3 integrin in osteoclasts during developmental stages.

Integrin signals reportedly crosstalk with growth factor signals. When osteoclasts are stimulated with growth factors, such as M-CSF and hepatocyte growth factor, small G proteins such as Rac, Rho, and Cdc42, are activated [51]. Activation of these small G proteins is not observed in *β3 integrin*-deficient osteoclasts or in wild-type osteoclasts cultured in suspension. These results indicate that adhesion-induced activation of integrin signaling promotes growth factor signaling. Furthermore, the mechanisms by which integrin signaling and c-Src promote osteoclast activity have been studied [52]. The tyrosine kinase Syk forms a complex with αvβ3 integrin, c-Src, and immunoreceptor tyrosine-based activation motif proteins such as DNAX activation protein of 12 kDa and FcRγ [53]. This complex activates the small G protein Rac to induce the bone-resorbing activity of osteoclasts.

Dynamin, a large GTPase involved in endocytosis, also plays roles in actin-ring formation in osteoclasts to bind a complex of c-Src, c-Cbl, and Pyk2 ([54], Fig. 1c). In fact, dynamin reportedly regulates the actin cytoskeleton in several cell types [55] and co-localizes with actin in osteoclasts [56, 57]. Furthermore, osteoclasts overexpressing dynamin K44A, a mutant form deficient in GTP binding, have decreased bone-resorbing activity [56]. Treatment of osteoclasts with dynasore, a GTPase inhibitor of dynamin, causes actin rings to rapidly disappear within 30 min [58]. Dynamin maintains podosome turnover by increasing Y402 dephosphorylation, which is required for Pyk2 activation [57]. These findings suggest that dynamin regulates the remodeling of podosomes to form actin rings in osteoclasts. The more precise mechanism by which dynamin regulates bone-resorbing activity of osteoclasts needs to clarify in future.

## Rac, Cdc42, and Rho

**Fig. 2:**
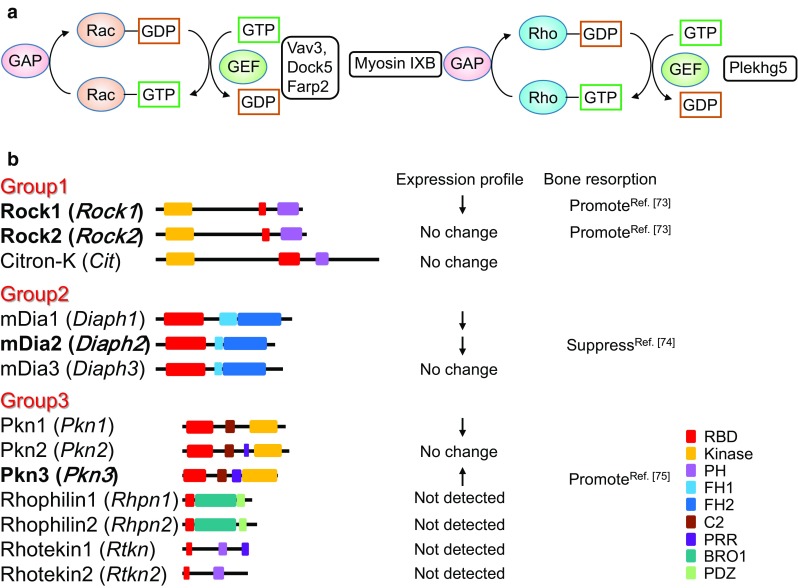
**a** Regulation of Rac and Rho activities. Rac GEFs and a Rho GEF involved in osteoclast function are indicated. *GEF* guanine nucleotide exchange factor, *GAP* GTPase-activating protein. **b** Rho effectors. Gene names are shown in parentheses. The downward arrows mean decreased expression during osteoclast differentiation, and the upward arrows mean increased expression. Regulation of bone resorption by Rho is shown with a reference number. *RBD* Rho-binding domain, *PH* Pleckstrin homology, *FH* Formin homology, *BRO1* BCK1-like resistance to osmotic shock protein 1, *PDZ* PSD95/Drosophila disks large/ZO-1, *PSD95* post-synaptic density 95, *ZO-1* Zonula occludens-1

Small G proteins are converted from the GDP-bound (inactive) form to the GTP-bound (active) form by a guanine nucleotide exchange factor (GEF) (Fig. 2a). Activated small G proteins become inactive by their own GTPase activity. GTPase-activating protein (GAP) promotes inactivation by increasing the GTPase activity of small G proteins [59]. The role of Rac GEFs in osteoclastic bone resorption has been reported [60–63]. Mice lacking the Rac GEF Vav3 have increased bone mass [60]. Osteoclasts derived from *Vav3*-deficient mice have suppressed activation of Rac by RANKL, M-CSF, and adhesion. Therefore, the formation of actin rings and resorption cavities are also impaired in *Vav3*-deficient osteoclasts. Although osteoclasts deficient in *Dock5*, another Rac GEF, fail to form actin rings and resorption cavities in in vitro experiments, bone mass is mildly increased in *Dock5*-deficient mice (approximately 20% increased from control litters) compared with Vav3-deficient mice (approximately 300% increase from control litters) [60, 62]. This finding suggests that Vav3, but not Dock5 is mainly involved in bone-resorbing activity of osteoclasts.

Crk-associated substrate (p130^Cas^), an adapter protein, is phosphorylated by c-Src [64]. Osteoclast-specific *p130*^*Cas*^-deficient mice have high bone mass from impaired bone-resorbing activity [63]. Mechanistically, osteoclasts lacking p130^Cas^ have impaired interactions among c-Src, Pyk2, and Dock5, which decreases Rac activity and impairs the formation of actin rings and resorption cavities. FERM, ARH/RhoGEF, and pleckstrin domain protein (FARP) 2, a Rac1-specific GEF, is also expressed in osteoclasts [61]. Osteoclasts that express FARP2 lacking a GEF domain (a dominant negative form) have impaired the formation of actin rings and resorption cavities, suggesting that FARP2 is also required for bone-resorbing activity of osteoclasts. In contrast to Vav3, FARP2 regulates the localization of active Rac1, but not the activation of Rac1 in osteoclasts. FARP2 negatively regulates phosphorylation of β3 integrin to reduce the adhesion activity [61]. Thus, FARP2 is involved in podosome rearrangement in osteoclasts and enhances bone-resorbing activity of osteoclasts.

Croke et al. [65] generated osteoclast-specific *Rac1; Rac2*^−/−^ double knockout mice (*Rac* DKO mice) by crossing *Rac1*^fl/fl^; *Rac2*^−/−^ mice with *LysM* Cre mice or with *Cathepsin K* Cre mice. Both *Rac* DKO mice using *LysM* Cre (LysM *Rac* DKO) and using *Cathepsin K* Cre (*Ctsk Rac* DKO) have an osteopetrotic phenotype. Osteoclasts formed from *LysM Rac* DKO fail to form actin rings and resorption cavities, but osteoclasts formed from *Ctsk Rac* DKO have normal bone-resorbing activity in vitro. Wang et al. [66] also generated *Rac* DKO mice by crossing *Rac2*^−/−^ and *Rac1*^fl/fl^ with *LysM* Cre mice to analyze the bone phenotype. In contrast to Croke’s report, the mice have a mild increase in bone mass and impaired bone-resorbing activity in osteoclasts. Furthermore, RANKL-induced osteoclast formation was impaired in cultures of osteoclast precursors derived from *Rac* DKO mice. These studies reveal the importance of Rac in the bone-resorbing activity of osteoclasts.

Osteoclast-specific *Cdc42*-deficient mice also have increased bone mass because of the decreased bone-resorbing activity of osteoclasts [67]. Actin-ring formation is slower in osteoclasts derived from *Cdc42*-deficient mice. Elongation and branching of F-actin occur via WASP, N-WASP, and the Arp2/3 complex, which are downstream of Cdc42 activity [29]. These findings suggest that Cdc42 plays critical roles in the formation of podosome cores in osteoclasts.

In contrast to Rac and Cdc42, roles of Rho in bone-resorbing activity of osteoclasts have not been established. When C3 exoenzyme, an inhibitor of Rho, is added to osteoclast cultures, actin rings disappear [68], which suggests that Rho is necessary for actin-ring formation in osteoclasts. In contrast, overexpression of a constitutively active form of Rho reduces actin-ring formation in osteoclasts [69]. Furthermore, when Pleckstrin homology (PH) domain-containing family G 5 (Plekhg5), a Rho GEF, is knocked down in osteoclasts, bone-resorbing activity is impaired [70]. These studies suggest that the activation of Rho may be tightly regulated in a narrow optimal window to promote the bone-resorbing activity of osteoclasts. Myosin IXB is a GAP for Rho in osteoclasts [71]. When myosin IXB is knocked down, the activity of Rho but not Rac slightly increases in osteoclasts. siRNA-mediated knockdown of *Myosin IXB* inhibits the formation of podosome belts and increases actin rings in osteoclasts cultured on glass. Interestingly, osteoclasts knocked down for *Myosin IXB* cultured on dentin slices which have impaired bone-resorbing activity, even though actin rings are formed normally. These osteoclasts have decreased phosphorylation of tyrosine residues necessary for c-Src activity and abnormal localization of c-Src, which suggests that Rho regulates the activity and localization of c-Src.

**Fig. 3:**
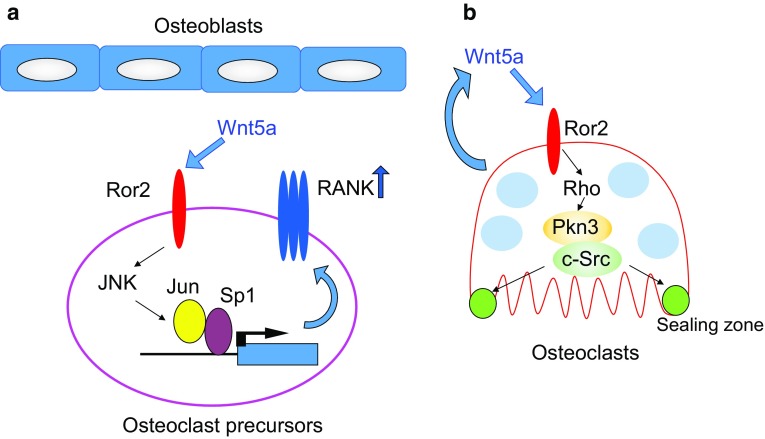
**a** The role of Wnt5a-Ror2 signaling in osteoclast differentiation. Wnt5a-Ror2 signaling increases the expression of RANK in osteoclast precursor cells through the activation of JNK, thereby promoting osteoclast differentiation. **b** Role of Wnt5a-Ror2 signaling in osteoclast function. Wnt5a-Ror2 signaling activates Rho and then promotes the activity of c-Src in a Pkn3-dependent manner. This signaling pathway enhances the bone-resorbing activity of osteoclasts

Because various effector molecules are activated downstream of Rho, regulation of Rho may be required for the bone-resorbing activity of osteoclasts. There are 13 Rho effectors that bind active Rho ([72], Fig. 3b), and they are classified into three groups: Group 1 contains Rho-associated, coiled-coil containing protein kinase (Rock) 1, Rock2, and citron-K, which are serine/threonine kinases. Group 2 contains mammalian homolog of *Drosophila* Diaphanous (mDia) 1–3, which have formin homology (FH) 1, 2 domains. mDia1, mDia2, and mDia3 are involved in actin elongation. Group 3 contains protein kinase N (Pkn) 1–3, which are serine/threonine kinases. Rhophilins and Rhotekins are involved in protein–protein interactions, which have a Rho-binding domain (RBD) and PSD95, Disks large, ZO-1 (PDZ) or PH domains, but no kinase domain. Rock1 and Rock2 positively control bone resorption by recruiting CD44, an osteopontin receptor, to the plasma membrane of osteoclasts [73]. On the other hand, mDia2 negatively regulates bone resorption by promoting deacetylation of tubulin through histone deacetylase (HDAC) 6 [74]. The expression of *Pkn3* increases during osteoclast differentiation and *Pkn3*-deficient mice have high bone mass because of decreased bone resorption [75]. Multiple effector signals may cooperate or counteract each other downstream of Rho. Taken together, Rho may regulate osteoclast activity, but further studies are needed to clarify the role of Rho in osteoclast function.

## Non-canonical Wnt signaling pathways

Wnt proteins are involved in the development and homeostasis of various organs through β-catenin-dependent and β-catenin-independent signaling [76–81]. There are 19 ligands involved in Wnt signaling in human and mouse. The ligands bind to frizzled receptors and the co-receptor low-density lipoprotein receptor-related protein (LRP) 5/6, and then, β-catenin-dependent canonical signaling pathways are activated [76, 79]. This signaling induces cytosolic accumulation and nuclear translocation of β-catenin. Nuclear β-catenin and T cell factor/lymphoid enhancer factor induce the transcription of target genes.

On the other hand, Wnt5a, a typical non-canonical Wnt ligand, binds to receptor tyrosine kinase-like orphan receptor (Ror) 1/2 and activates β-catenin-independent signaling pathways such as the planar cell polarity (PCP) pathway and calcium pathway [77, 78]. In the PCP pathway, Rho, Rac, and Cdc42 are activated. Frizzled is associated with disheveled and disheveled-associated activator of morphogenesis (Daam) to activate Rho, Rac, and Cdc42. In the calcium pathway, intracellular calcium increases through receptor-coupled G proteins and phospholipase C.

Wnt5a is secreted from osteoblasts and binds to the Ror2 receptor in osteoclast precursor cells, which promotes RANKL-induced osteoclast differentiation [80]. Mechanistically, Wnt5a activates c-Jun N-terminal kinase (JNK) in osteoclast precursor cells through Ror2-mediated signaling, which enhances the expression of RANK (Fig. 3a). Expression of Wnt5a and Ror2 markedly increases during osteoclast differentiation [75], which suggests that Wnt5a regulates the function of mature osteoclasts through Ror2 receptors. Osteoclast precursor cells prepared from the fetal liver of *Wnt5a*-deficient mice with M-CSF and RANKL have impaired bone-resorbing activity due to defects in actin-ring formation. These results suggest that Wnt5a secreted from osteoclasts autonomously promotes bone-resorbing activity.

To clarify the role of Ror2 signaling in osteoclast function, bone from osteoclast-specific *Ror2*-deficient (*Ror2*^ΔOCL/ΔOCL^) mice (by crossing *Ror2*^fl/fl^ mice with *Cathepsin K*-Cre mice) was analyzed. *Ror2*^ΔOCL/ΔOCL^ have high bone mass because of the impaired bone-resorbing activity of osteoclasts. Wnt5a activates both Rac and Rho, but overexpression of a constitutively active form of RhoA but not Rac1 rescues the impaired bone-resorbing activity of *Ror2*^ΔOCL/ΔOCL^ osteoclasts. These findings suggest that Wnt5a-Ror2 signaling activates Rho, which promotes the bone-resorbing activity of osteoclasts. Daams 1 and 2 activate Rho downstream of frizzled and disheveled [75, 81], and shRNA-mediated knockdown of *Daam2* suppresses the bone-resorbing activity of osteoclasts. These findings suggest that Wnt5a-Ror2 signaling activates Rho through Daam2 to promote the bone-resorbing activity of osteoclasts.

Expression of Pkn3 markedly increases in osteoclasts, and actin-ring formation and bone-resorbing activity are lower in osteoclasts derived from *Pkn3*-deficient mice. Similar to *Ror2*^ΔOCL/ΔOCL^ mice, *Pkn3*-deficient mice have increased bone mass due to impaired bone resorption, but not increased bone formation. Pkn3 is associated with c-Src and Pyk2 in a Ror2- and Daam2-dependent manner. In addition, the kinase activity of c-Src decreases in *Ror2*^ΔOCL/ΔOCL^ and *Pkn3*-deficient osteoclasts. The proline-rich region of Pkn3 is necessary for binding between Pkn3 and c-Src, and for the bone-resorbing activity of osteoclasts. Furthermore, Pkn3 lacking the kinase domain bound to c-Src but failed to rescue the impaired bone-resorbing activity of *Pkn3*-deficient osteoclasts. This finding suggests that the kinase domain is also required for the activation of c-Src by Pkn3. The mechanism by which Pkn3 activates c-Src in osteoclasts needs to clarify in future. Taken together, Wnt5a-Ror2 signaling activates Rho via Daam2 in osteoclasts. Activation of Rho promotes the formation Pkn3, c-Src, and Pyk2 complexes, and increases c-Src activity in osteoclasts, thereby promoting actin-ring formation and bone resorption (Fig. 3b). Thus, Pkn3 represents a therapeutic target for osteoporosis and inflammatory bone diseases such as periodontal disease and rheumatoid arthritis.

## Conclusion

Many molecules and signaling pathways involved in the formation of actin rings and bone-resorbing activity of osteoclasts have been identified. Bone resorption by osteoclasts is a tightly regulated multistep process. Mouse genetic approaches have revealed the role of Rac in osteoclast function, and several Rac GEFs, such as Vav3, Dock5, and FARP2, are involved. However, it is still not clear why so many molecules are necessary to activate Rac during bone resorption. Rho is also activated by many signals, and further studies are needed to clarify how Rac cooperates with Rho to organize podosomes in osteoclasts. These studies may lead to the development of new antiresorptive drugs and will clarify when and where molecules are activated during cytoskeletal remodeling in osteoclasts. The development of multiphoton fluorescence microscopes and new probes, such as LifeAct-GFP [82], will enable real-time analysis of actin dynamics and other molecules.

## References

[1] Takeda S, Elefteriou F, Karsenty G (2003). Common endocrine control of body weight, reproduction, and bone mass. Annu Rev Nutr.

[2] Zaidi M (2007). Skeletal remodeling in health and disease. Nat Med.

[3] Eastell R, O’Neill TW, Hofbauer LC, Langdahl B, Reid IR, Gold DT, Cummings SR (2016). Postmenopausal osteoporosis. Nat Rev Dis Primers.

[4] Okamoto K, Nakashima T, Shinohara M, Negishi-Koga T, Komatsu N, Terashima A, Sawa S, Nitta T, Takayanagi H (2017). Osteoimmunology: the conceptual framework unifying the immune and skeletal systems. Physiol Rev.

[5] Algate K, Haynes DR, Bartold PM, Crotti TN, Cantley MD (2016). The effects of tumour necrosis factor-α on bone cells involved in periodontal alveolar bone loss; osteoclasts, osteoblasts and osteocytes. J Periodontal Res.

[6] Baron R, Ferrari S, Russell RG (2011). Denosumab and bisphosphonates: different mechanisms of action and effects. Bone.

[7] Rachner TD, Khosla S, Hofbauer LC (2011). Osteoporosis: now and the future. Lancet.

[8] Xu XL, Gou WL, Wang AY, Wang Y, Guo QY, Lu Q, Lu SB, Peng J (2013). Basic research and clinical applications of bisphosphonates in bone disease: what have we learned over the last 40 years?. J Transl Med.

[9] Pollard TD, Cooper JA (2009). Actin, a central player in cell shape and movement. Science.

[10] Parsons JT, Horwitz AR, Schwartz MA (2010). Cell adhesion: integrating cytoskeletal dynamics and cellular tension. Nat Rev Mol Cell Biol.

[11] Elosegui-Artola A, Trepat X, Roca-Cusachs P (2018). Control of mechanotransduction by molecular clutch dynamics. Trends Cell Biol.

[12] Krause M, Gautreau A (2014). Steering cell migration: lamellipodium dynamics and the regulation of directional persistence. Nat Rev Mol Cell Biol.

[13] Skau CT, Waterman CM (2015). Specification of architecture and function of actin structures by actin nucleation factors. Annu Rev Biophys.

[14] van den Dries K, Bolomini-Vittori M, Cambi A (2014). Spatiotemporal organization and mechanosensory function of podosomes. Cell Adhes Migr.

[15] Linder S, Wiesner C (2015). Tools of the trade: podosomes as multipurpose organelles of monocytic cells. Cell Mol Life Sci.

[16] Mak AS (2011). p53 regulation of podosome formation and cellular invasion in vascular smooth muscle cells. Cell Adhes Migr.

[17] Parekh A, Weaver AM (2016). Regulation of invadopodia by mechanical signaling. Exp Cell Res.

[18] Spuul P, Daubon T, Pitter B, Alonso F, Fremaux I, Kramer I, Montanez E, Génot E (2016). VEGF-A/Notch-induced podosomes proteolyse basement membrane collagen-IV during retinal sprouting angiogenesis. Cell Rep.

[19] Destaing O, Ferguson SM, Grichine A, Oddou C, De Camilli P, Albiges-Rizo C, Baron R (2013). Essential function of dynamin in the invasive properties and actin architecture of v-Src induced podosomes/invadosomes. PLoS One.

[20] Kuo SL, Chen CL, Pan YR, Chiu WT, Chen HC (2018). Biogenesis of podosome rosettes through fission. Sci Rep.

[21] Boyce BF, Yoneda T, Lowe C, Soriano P, Mundy GR (1992). Requirement of pp60c-src expression for osteoclasts to form ruffled borders and resorb bone in mice. J Clin Investig.

[22] Suda T, Takahashi N, Udagawa N, Jimi E, Gillespie MT, Martin TJ (1999). Modulation of osteoclast differentiation and function by the new members of the tumor necrosis factor receptor and ligand families. Endocr Rev.

[23] Boyle WJ, Simonet WS, Lacey DL (2003). Osteoclast differentiation and activation. Nature.

[24] Feng X, Teitelbaum SL (2013). Osteoclasts: new insights. Bone Res.

[25] Søe K, Delaissé J-M (2017). Time-lapse reveals that osteoclasts can move across the bone surface while resorbing. J Cell Sci.

[26] Takito J, Inoue S, Nakamura M (2018). The sealing zone in osteoclasts: a self-organized structure on the bone. Int J Mol Sci..

[27] Kikuta J, Wada Y, Kowada T, Wang Z, Sun-Wada GH, Nishiyama I, Mizukami S, Maiya N, Yasuda H, Kumanogoh A, Kikuchi K, Germain RN, Ishii M (2013). Dynamic visualization of RANKL and Th17-mediated osteoclast function. J Clin Investig.

[28] Georgess D, Machuca-Gayet I, Blangy A, Jurdic P (2014). Podosome organization drives osteoclast-mediated bone resorption. Cell Adhes Migr.

[29] Soysa NS, Alles N (2016). Osteoclast function and bone-resorbing activity: an overview. Biochem Biophys Res Commun.

[30] Nesbitt SA, Horton MA (1997). Trafficking of matrix collagens through bone-resorbing osteoclasts. Science.

[31] Salo J, Lehenkari P, Mulari M, Metsikkö K, Väänänen HK (1997). Removal of osteoclast bone resorption products by transcytosis. Science.

[32] Takahashi N, Udagawa N, Kobayashi Y, Suda T (2007). Generation of osteoclasts in vitro, and assay of osteoclast activity. Methods Mol Med.

[33] Nakayama T, Mizoguchi T, Uehara S, Yamashita T, Kawahara I, Kobayashi Y, Moriyama Y, Kurihara S, Sahara N, Ozawa H, Udagawa N, Takahashi N (2011). Polarized osteoclasts put marks of tartrate-resistant acid phosphatase on dentin slices–a simple method for identifying polarized osteoclasts. Bone.

[34] Geblinger D, Addadi L, Geiger B (2010). Nano-topography sensing by osteoclasts. J Cell Sci.

[35] van den Dries K, van Helden SF, te Riet J, Diez-Ahedo R, Manzo C, Oud MM, van Leeuwen FN, Brock R, Garcia-Parajo MF, Cambi A, Figdor CG (2012). Geometry sensing by dendritic cells dictates spatial organization and PGE(2)-induced dissolution of podosomes. Cell Mol Life Sci.

[36] Baranov M, Ter Beest M, Reinieren-Beeren I, Cambi A, Figdor CG, van den Bogaart G (2014). Podosomes of dendritic cells facilitate antigen sampling. J Cell Sci.

[37] Woo JT, Kawatani M, Kato M, Shinki T, Yonezawa T, Kanoh N, Nakagawa H, Takami M, Lee KH, Stern PH, Nagai K, Osada H (2006). Reveromycin A, an agent for osteoporosis, inhibits bone resorption by inducing apoptosis specifically in osteoclasts. Proc Natl Acad Sci USA.

[38] Mizutani K, Miki H, He H, Maruta H, Takenawa T (2002). Essential role of neural Wiskott–Aldrich syndrome protein in podosome formation and degradation of extracellular matrix in src-transformed fibroblasts. Cancer Res.

[39] Hurst IR, Zuo J, Jiang J, Holliday LS (2004). Actin-related protein 2/3 complex is required for actin ring formation. J Bone Miner Res.

[40] Calle Y, Jones GE, Jagger C, Fuller K, Blundell MP, Chow J, Chambers T, Thrasher AJ (2004). WASp deficiency in mice results in failure to form osteoclast sealing zones and defects in bone resorption. Blood.

[41] Isaac BM, Ishihara D, Nusblat LM, Gevrey JC, Dovas A, Condeelis J, Cox D (2010). N-WASP has the ability to compensate for the loss of WASP in macrophage podosome formation and chemotaxis. Exp Cell Res.

[42] Tehrani S, Faccio R, Chandrasekar I, Ross FP, Cooper JA (2006). Cortactin has an essential and specific role in osteoclast actin assembly. Mol Biol Cell.

[43] Soriano P, Montgomery C, Geske R, Bradley A (1991). Targeted disruption of the c-src proto-oncogene leads to osteopetrosis in mice. Cell.

[44] Destaing O, Sanjay A, Itzstein C, Horne WC, Toomre D, De Camilli P, Baron R (2008). The tyrosine kinase activity of c-Src regulates actin dynamics and organization of podosomes in osteoclasts. Mol Biol Cell.

[45] Gil-Henn H, Destaing O, Sims NA, Aoki K, Alles N, Neff L, Sanjay A, Bruzzaniti A, De Camilli P, Baron R, Schlessinger J (2007). Defective microtubule-dependent podosome organization in osteoclasts leads to increased bone density in Pyk2(−/−) mice. J Cell Biol.

[46] Sanjay A, Houghton A, Neff L, DiDomenico E, Bardelay C, Antoine E, Levy J, Gailit J, Bowtell D, Horne WC, Baron R (2001). Cbl associates with Pyk2 and Src to regulate Src kinase activity, alpha(v)beta(3) integrin-mediated signaling, cell adhesion, and osteoclast motility. J Cell Biol.

[47] Zou W, Izawa T, Zhu T, Chappel J, Otero K, Monkley SJ, Critchley DR, Petrich BG, Morozov A, Ginsberg MH, Teitelbaum SL (2013). Talin1 and Rap1 are critical for osteoclast function. Mol Cell Biol.

[48] Fukunaga T, Zou W, Warren JT, Teitelbaum SL (2014). Vinculin regulates osteoclast function. J Biol Chem.

[49] Zambonin-Zallone A, Teti A, Grano M, Rubinacci A, Abbadini M, Gaboli M, Marchisio PC (1989). Immunocytochemical distribution of extracellular matrix receptors in human osteoclasts: a beta 3 integrin is colocalized with vinculin and talin in the podosomes of osteoclastoma giant cells. Exp Cell Res.

[50] Quinn JM, Athanasou NA, McGee JO (1991). Extracellular matrix receptor and platelet antigens on osteoclasts and foreign body giant cells. Histochemistry.

[51] Faccio R, Novack DV, Zallone A, Ross FP, Teitelbaum SL (2003). Dynamic changes in the osteoclast cytoskeleton in response to growth factors and cell attachment are controlled by beta3 integrin. J Cell Biol.

[52] Zou W, Teitelbaum SL (2010). Integrins, growth factors, and the osteoclast cytoskeleton. Ann N Y Acad Sci.

[53] Zou W, Kitaura H, Reeve J, Long F, Tybulewicz VL, Shattil SJ, Ginsberg MH, Ross FP, Teitelbaum SL (2007). Syk, c-Src, the alphavbeta3 integrin, and ITAM immunoreceptors, in concert, regulate osteoclastic bone resorption. J Cell Biol.

[54] Bruzzaniti A, Neff L, Sanjay A, Horne WC, De Camilli P, Baron R (2005). Dynamin forms a Src kinase-sensitive complex with Cbl and regulates podosomes and osteoclast activity. Mol Biol Cell.

[55] Menon M, Schafer DA (2013). Dynamin: expanding its scope to the cytoskeleton. Int Rev Cell Mol Biol.

[56] Ochoa GC, Slepnev VI, Neff L, Ringstad N, Takei K, Daniell L, Kim W, Cao H, McNiven M, Baron R, De Camilli P (2000). A functional link between dynamin and the actin cytoskeleton at podosomes. J Cell Biol.

[57] Bruzzaniti A, Neff L, Sandoval A, Du L, Horne WC, Baron R (2009). Dynamin reduces Pyk2 Y402 phosphorylation and SRC binding in osteoclasts. Mol Cell Biol.

[58] Thirukonda GJ, Uehara S, Nakayama T, Yamashita T, Nakamura Y, Mizoguchi T, Takahashi N, Yagami K, Udagawa N, Kobayashi Y (2016). The dynamin inhibitor dynasore inhibits bone resorption by rapidly disrupting actin rings of osteoclasts. J Bone Miner Metab.

[59] Lawson CD, Burridge K (2014). The on-off relationship of Rho and Rac during integrin-mediated adhesion and cell migration. Small GTPases.

[60] Faccio R, Teitelbaum SL, Fujikawa K, Chappel J, Zallone A, Tybulewicz VL, Ross FP, Swat W (2005). Vav3 regulates osteoclast function and bone mass. Nat Med.

[61] Takegahara N, Kang S, Nojima S, Takamatsu H, Okuno T, Kikutani H, Toyofuku T, Kumanogoh A (2010). Integral roles of a guanine nucleotide exchange factor, FARP2, in osteoclast podosome rearrangements. FASEB J.

[62] Vives V, Laurin M, Cres G, Larrousse P, Morichaud Z, Noel D, Côté JF, Blangy A (2011). The Rac1 exchange factor Dock5 is essential for bone resorption by osteoclasts. J Bone Miner Res.

[63] Nagai Y, Osawa K, Fukushima H, Tamura Y, Aoki K, Ohya K, Yasuda H, Hikiji H, Takahashi M, Seta Y, Seo S, Kurokawa M, Kato S, Honda H, Nakamura I, Maki K, Jimi E (2013). p130Cas, Crk-associated substrate, plays important roles in osteoclastic bone resorption. J Bone Miner Res.

[64] Nakamura I, Jimi E, Duong LT, Sasaki T, Takahashi N, Rodan GA, Suda T (1998). Tyrosine phosphorylation of p130Cas is involved in actin organization in osteoclasts. J Biol Chem.

[65] Croke M, Ross FP, Korhonen M, Williams DA, Zou W, Teitelbaum SL (2011). Rac deletion in osteoclasts causes severe osteopetrosis. J Cell Sci.

[66] Wang Y, Lebowitz D, Sun C, Thang H, Grynpas MD, Glogauer M (2008). Identifying the relative contributions of Rac1 and Rac2 to osteoclastogenesis. J Bone Miner Res.

[67] Ito Y, Teitelbaum SL, Zou W, Zheng Y, Johnson JF, Chappel J, Ross FP, Zhao H (2010). Cdc42 regulates bone modeling and remodeling in mice by modulating RANKL/M-CSF signaling and osteoclast polarization. J Clin Investig.

[68] Zhang D, Udagawa N, Nakamura I, Murakami H, Saito S, Yamasaki K, Shibasaki Y, Morii N, Narumiya S, Takahashi N (1995). The small GTP-binding protein, rho p21, is involved in bone resorption by regulating cytoskeletal organization in osteoclasts. J Cell Sci.

[69] Ory S, Munari-Silem Y, Fort P, Jurdic P (2000). Rho and Rac exert antagonistic functions on spreading of macrophage-derived multinucleated cells and are not required for actin fiber formation. J Cell Sci.

[70] Iwatake M, Nishishita K, Okamoto K, Tsukuba T (2017). The Rho-specific guanine nucleotide exchange factor Plekhg5 modulates cell polarity, adhesion, migration, and podosome organization in macrophages and osteoclasts. Exp Cell Res.

[71] McMichael BK, Scherer KF, Franklin NC, Lee BS (2014). The RhoGAP activity of myosin IXB is critical for osteoclast podosome patterning, motility, and resorptive capacity. PLoS One.

[72] Thumkeo D, Watanabe S, Narumiya S (2013). Physiological roles of Rho and Rho effectors in mammals. Eur J Cell Biol.

[73] Chellaiah MA, Biswas RS, Rittling SR, Denhardt DT, Hruska KA (2003). Rho-dependent Rho kinase activation increases CD44 surface expression and bone resorption in osteoclasts. J Biol Chem.

[74] Destaing O, Saltel F, Gilquin B, Chabadel A, Khochbin S, Ory S, Jurdic P (2005). A novel Rho-mDia2-HDAC6 pathway controls podosome patterning through microtubule acetylation in osteoclasts. J Cell Sci.

[75] Uehara S, Udagawa N, Mukai H, Ishihara A, Maeda K, Yamashita T, Murakami K, Nishita M, Nakamura T, Kato S, Minami Y, Takahashi N, Kobayashi Y (2017). Protein kinase N3 promotes bone resorption by osteoclasts in response to Wnt5a-Ror2 signaling. Sci Signal.

[76] Nusse R (2008). Wnt signaling and stem cell control. Cell Res.

[77] Lai SL, Chien AJ, Moon RT (2009). Wnt/Fz signaling and the cytoskeleton: potential roles in tumorigenesis. Cell Res.

[78] Minami Y, Oishi I, Endo M, Nishita M (2010). Ror-family receptor tyrosine kinases in noncanonical Wnt signaling: their implications in developmental morphogenesis and human diseases. Dev Dyn.

[79] Nusse R, Clevers H (2017). Wnt/β-catenin signaling, disease, and emerging therapeutic modalities. Cell.

[80] Maeda K, Kobayashi Y, Udagawa N, Uehara S, Ishihara A, Mizoguchi T, Kikuchi Y, Takada I, Kato S, Kani S, Nishita M, Marumo K, Martin TJ, Minami Y, Takahashi N (2012). Wnt5a-Ror2 signaling between osteoblast-lineage cells and osteoclast precursors enhances osteoclastogenesis. Nat Med.

[81] Habas R, Kato Y, He X (2001). Wnt/Frizzled activation of Rho regulates vertebrate gastrulation and requires a novel Formin homology protein Daam1. Cell.

[82] Meddens MB, Pandzic E, Slotman JA, Guillet D, Joosten B, Mennens S, Paardekooper LM, Houtsmuller AB, van den Dries K, Wiseman PW, Cambi A (2016). Actomyosin-dependent dynamic spatial patterns of cytoskeletal components drive mesoscale podosome organization. Nat Commun.

